# Sepsis-Associated Delirium: A Narrative Review

**DOI:** 10.3390/jcm12041273

**Published:** 2023-02-06

**Authors:** Rina Tokuda, Kensuke Nakamura, Yudai Takatani, Chie Tanaka, Yutaka Kondo, Hiroyuki Ohbe, Hiroshi Kamijo, Kosuke Otake, Atsuo Nakamura, Hiroyasu Ishikura, Yu Kawazoe, J-STAD (Japan Sepsis Treatment and Diagnosis) Study Group

**Affiliations:** 1 Tajima Emergency and Critical Care Medical Center, Toyooka Public Hospital, Hyogo 668-8501, Japan; 2 Department of Emergency and Critical Care Medicine, Teikyo University Hospital, Tokyo 173-8606, Japan; 3 Department of Primary Care and Emergency Medicine, Kyoto University Hospital, Kyoto 606-8507, Japan; 4 Department of Emergency and Critical Care Medicine, Nippon Medical School Tama Nagayama Hospital, Tokyo 206-8512, Japan; 5 Department of Emergency and Critical Care Medicine, Juntendo University Urayasu Hospital, Chiba 279-0021, Japan; 6 Department of Clinical Epidemiology and Health Economics, School of Public Health, The University of Tokyo, Tokyo 113-8654, Japan; 7 Department of Emergency and Critical Care Medicine, Shinshu University Hospital, Nagano 390-0802, Japan; 8 Department of Emergency and Critical Care Center, Nippon Medical School Musashikosugi Hospital, Kanagawa 211-8533, Japan; 9 Department of Emergency and Critical Care Medicine, Iizuka City Hospital, Fukuoka 820-8505, Japan; 10 Department of Emergency and Critical Care Medicine, Faculty of Medicine, Fukuoka University, Fukuoka 814-0180, Japan; 11 Department of Emergency Critical Care Center, Sendai Medical Center, Miyagi 983-0045, Japan

**Keywords:** sepsis, delirium, COVID-19, post-intensive care syndrome

## Abstract

Delirium is characterized by an acutely altered mental status accompanied by reductions in cognitive function and attention. Delirium in septic patients, termed sepsis-associated delirium (SAD), differs in several specific aspects from the other types of delirium that are typically encountered in intensive care units. Since sepsis and delirium are both closely associated with increased morbidity and mortality, it is important to not only prevent but also promptly diagnose and treat SAD. We herein reviewed the etiology, pathogenesis, risk factors, prevention, diagnosis, treatment, and prognosis of SAD, including coronavirus disease 2019 (COVID-19)-related delirium. Delirium by itself not only worsens long-term prognosis, but it is also regarded as an important factor affecting the outcome of post-intensive care syndrome. In COVID-19 patients, the difficulties associated with adequately implementing the ABCDEF bundle (Assess, prevent, and manage pain; Both spontaneous awakening and breathing trials: Choice of analgesia and sedation; Delirium assess, prevent, and manage; Early mobility and exercise; Family engagement/empowerment) and the need for social isolation are issues that require the development of conventional care for SAD.

## 1. Introduction

Delirium is characterized by an acutely altered mental status accompanied by reductions in cognitive function and attention that fluctuates with time and occurs in 20~40% of critically ill patients admitted to the intensive care unit (ICU) [1,2,3]. Although a number of factors influence the pathophysiology of delirium, many have yet to be identified [4]. Sepsis, a life-threatening organ dysfunction caused by an abnormal host response to infection [5], is one of the most important and strongest risk factors for delirium [4,6,7]. Delirium in septic patients, termed sepsis-associated delirium (SAD), differs in several specific aspects from the other types of delirium typically encountered in the ICU. Since sepsis and delirium are both closely associated with increased morbidity and mortality [5,8,9], it is important to not only prevent but also promptly diagnose and treat SAD.

We herein review and discuss the etiology, pathogenesis, risk factors, prevention, diagnosis, treatment, and prognosis of SAD, including coronavirus disease 2019 (COVID-19)-related delirium.

## 2. Epidemiology

Delirium often occurs in hospitalized adults in general medical settings. In a systematic review conducted in 2020, the overall prevalence of delirium was 23% in acute adult inpatients [10]. An observational longitudinal study reported that delirium was rare among acute medical patients younger than 65 years old but was 10-fold more prevalent in those older than 75 years [11].

### 2.1. Subsyndromal Delirium

The prevalence of subsyndromal delirium (SSD), which does not meet the formal criteria for delirium, highly varies and depends on the definition used as well as the population being examined. In a large multicenter cohort study, SSD developed in more than 80% of patients at some point during their ICU stay [12].

### 2.2. Clinical Phenotypes of Delirium in the ICU

Delirium frequently develops in the ICU, and the prevalence of each phenotype defined by clinical risk factors has been examined. Among 1040 patients, 71% were diagnosed with delirium at some point during their ICU stay: sedative-associated, hypoxic, septic, metabolic, and unclassified delirium in 64, 56, 51, 25, and 22%, respectively [13].

### 2.3. SAD

SAD is one of the symptoms of sepsis-associated encephalopathy (SAE), which represents a wide spectrum of symptoms from sickness behavior and delirium to coma. A retrospective analysis of 2513 patients revealed that 53% had SAE, 19% of whom were diagnosed with delirium and 40% with coma [7].

### 2.4. COVID-19-Associated Delirium

In the post-COVID-19 pandemic era, a systemic review revealed that the prevalence of delirium was 24.3% in COVID-19 patients and 28.2% in those older than 65 years old. Furthermore, delirium developed in approximately 50% of patients in the ICU and in 26.3% of those in non-ICU settings [14].

## 3. Etiology and Pathophysiology of Delirium

**Figure 1:**
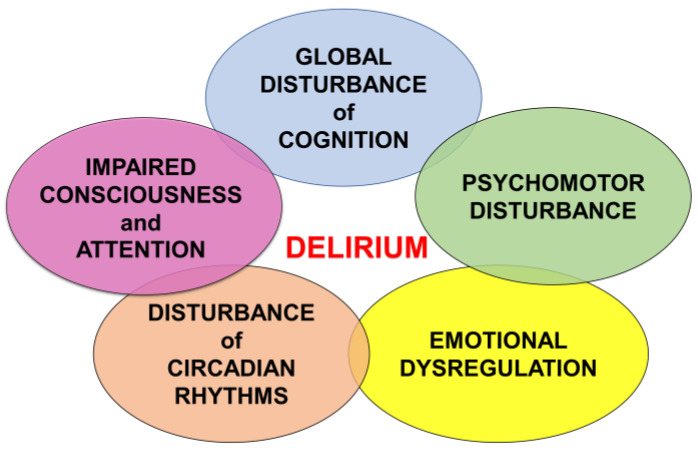
The clinical features of delirium.

Delirium is the most common neuropsychiatric syndrome encountered by clinicians dealing with older adults and the medically ill [15]. The clinical features of delirium include five core domains: cognitive deficits, attentional deficits, circadian rhythm dysregulation, emotional dysregulation, and alterations in psychomotor functioning, which are characterized by the acute onset of symptoms with a fluctuating course [16] (Figure 1).

**Figure 2:**
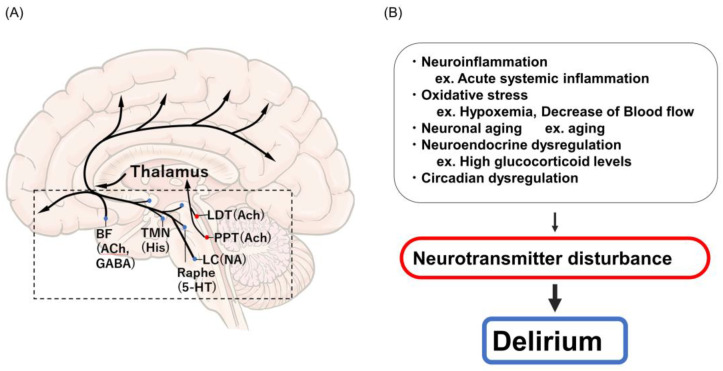
(**A**) Neurotransmitter of consciousness and arousal system. (**B**) Etiological factors of delirium. 5-HT, 5-hydroxytryptamine; ACh, acetylcholine; BF, basal forebrain; His, histamine; LC, locus coeruleus; LDT, laterodorsal tegmental nucleus; NA, noradrenaline; PPT, pedunculopontine tegmentum; TMN, tuberomammillary nucleus.

Despite its clinical impact, the pathophysiological mechanisms underlying delirium have not yet been elucidated in detail [17]. Central cholinergic deficiency [18], excess dopamine, inflammation, chronic stress, hypoxemia, impaired blood flow and tissue perfusion, and impaired metabolism (hyponatremia, hypernatremia, and hypoglycemia) have been proposed as the pathophysiological mechanisms responsible for delirium [19,20]. Anatomically, cholinergic pathways have widespread interconnections in the brain, and acetylcholine plays an extensive role in sensory and cognitive inputs; therefore, irregularities in these brain functions cause the core symptoms of delirium. The reduced availability of acetylcholine, the excess release of dopamine, norepinephrine, and glutamine, and changes in serotonin, histamine, and gamma-aminobutyric acid levels in the brain are associated with delirium and interact with other important neurotransmitter of consciousness and arousal systems (Figure 2A,B).

Etiological factors for the development of delirium include neuronal aging, neuroinflammation, oxidative stress, neuroendocrine dysregulation, and circadian dysregulation. An older age has been identified as an independent risk factor for delirium among hospitalized, medically ill, older patients [21]. Inflammatory cytokines and mediators in the central nervous system (e.g., C-reactive protein, interleukin [IL]-6, tumor necrosis factor alpha, IL-1RA, IL-10, and IL-8) contribute to the neuronal and synaptic dysfunctions and subsequent neurobehavioral and cognitive symptoms that are characteristic of delirium [22,23]. Oxidative stress and/or antioxidant deficiencies may increase damage to cerebral tissue, leading to cognitive decline and potentially the development of persistent delirium [24]. Increases in glucocorticoid levels in response to acute or chronic stress also result in delirium as a physiological reaction [25]. Moreover, disturbances in the circadian cycle have been implicated in the development of delirium [26]. The factors contributing to delirium interact with each other and cause neurotransmitter dysregulation and network dysconnectivity, which is called the systems integration failure hypothesis [27]. The combined effects of precipitant and substrate factors result in the final common pathways associated with delirium, including various phenotypic presentations.

## 4. Etiology and Pathophysiology of SAD

The mechanisms underlying SAD currently remain unclear; however, neurological decline is observed in patients with chronic sepsis. SAD has been attributed to a combination of neuroinflammation and disturbances in brain perfusion, the blood–brain barrier, and neurotransmission [4]. Neuroinflammation persists for several days to several months after the onset of sepsis and may lead to SAD, which often results in a cognitive-deficient, depressed mental status and neurological dysfunction even after the recovery of sepsis.

During sepsis, endothelial cells are activated by increased systemic levels of the proinflammatory cytokines IL-1β, IL-6, and TNF-α. Endothelial cell activation also promotes the production of reactive oxygen species. The blood–brain barrier is permeable to proinflammatory cytokines and reactive oxygen species, which may contribute to the development of SAD. Furthermore, endothelial cell activation has been associated with an increase in endothelial permeability, which impairs the microcirculation in the brain during sepsis [28,29]. Many factors are involved in SAD, and most of the underlying mechanisms proposed to date have been derived from the findings of animal studies or cell culture experiments.

Ischemic or hemorrhagic lesions have also been suggested to cause SAD [30]. In a comparative study, patients with septic shock were examined using immunocytochemistry, which revealed multiple ischemic lesions (100%), hemorrhage (26%), hypercoagulability (9%), micro-abscesses (9%), and multifocal necrotizing leukoencephalopathy (9%) [6]. Therefore, sepsis causes diffuse brain damage, which may lead to the development of SAD.

## 5. Risk Factors and Predictive Indicators of SAD

Previous studies have reported risk factors for SAD and SAE using different definitions and populations. In a landmark single-center study conducted on 50 non-sedated septic patients, 54% had SAE accompanied by bacteremia, elevated serum urea nitrogen and bilirubin levels, increased acute physiology and chronic health evaluation II (APACHE II) scores, and a higher incidence of renal failure [31]. In a single-center study on 232 patients with sepsis in the ICU, 18% had SAE, higher APACHE II scores, metabolic disturbances, the site of infection, and the type of microorganism related to the presence of SAE [32]. In another single-center study on 175 patients with sepsis in the ICU, among whom 61% had SAD, risk factors for SAD included age ≥65 years, dependent activity and high nursing needs, a low level of consciousness, tachypnea, thrombocytopenia, the use of vasopressors/inotropes, and decreased albumin levels [33]. These single-center studies had a number of limitations, including a small sample size, a single-center design, and no adjustments for important risk factors for the ICU. In a multicenter study on 2513 sepsis patients in 12 ICUs, among whom 53% had SAE, a multivariate analysis identified an older age, a history of chronic alcohol abuse, neurological disease, pre-existing cognitive dysfunction, the long-term use of psychoactive drugs, acute renal failure, hypoglycemia, hyperglycemia, hypercapnia, hypernatremia, and Staphylococcus aureus bacteremia as independent risk factors for SAE [7]. Collectively, these findings indicate that some risk factors for SAD and SAE are modifiable, whereas others are not. Although these risk factors may play a key role in the pathophysiology of SAD or SAE, it currently remains unclear whether there is a true causal relationship (Table 1).

## 6. Subtypes of Delirium

Delirium is classified into three motor subtypes: hyperactive, hypoactive, and mixed delirium [34]. Hyperactive delirium is characterized by agitation, hypervigilance, and hallucinations, whereas hypoactive delirium is characterized by stupor, psychomotor retardation, and lethargy. In mixed delirium, the patient shifts between hyperactive and hypoactive delirium.

**Figure 3:**
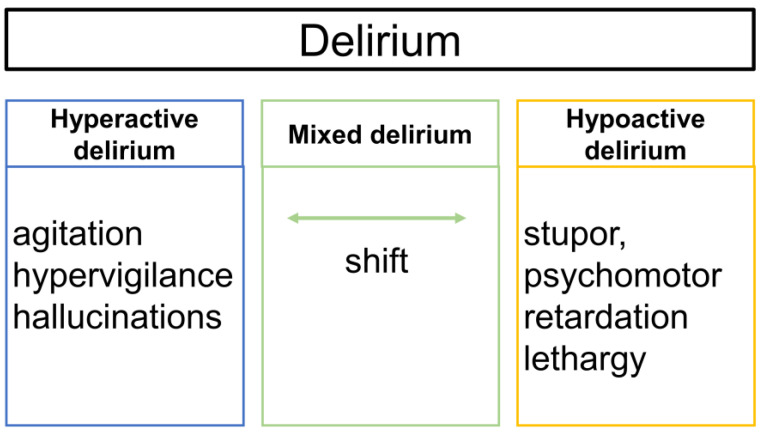
Subtypes of delirium.

A recent study reported that hypoactive delirium accounted for 50.3% of cases of delirium in the ICU, that it was the most common delirium motor subtype, and that patients with mixed delirium had the longest duration of delirium, longest ICU and hospital stays, and highest ICU and hospital mortality rates [35]. Furthermore, without the use of delirium screening tools, delirium may be overlooked in approximately 75% of patients with delirium in the ICU, particularly hypoactive delirium [36] (Figure 3).

## 7. Diagnosis and Severity Evaluation

The latest clinical practice guidelines on delirium recommend regular assessments for delirium using the Confusion Assessment Method-ICU (CAM-ICU) and Intensive Care Delirium Screening Checklist (ICDSC), which have both been widely validated and frequently used in the assessment and diagnosis of delirium in the ICU [37]. CAM-ICU and ICDSC may be used by non-psychiatric ICU personnel to diagnose complications quickly and reliably and may be adopted when a patient is unable to speak due to endotracheal intubation.

A recent systematic review and meta-analysis of studies on ICU patients reported that CAM-ICU had a pooled sensitivity of 80% and specificity of 95.9%, while ICDSC had a pooled sensitivity of 74% and specificity of 81.9% [38]. CAM-ICU is superior to ICDSC for excluding patients without ICU delirium and detecting delirium in medical ICUs and in patients receiving ventilation.

Previous studies validated the scales used to assess the severity of delirium, including CAM-ICU-7, which is scored from CAM-ICU and the Richmond Agitation–Sedation Scale. CAM-ICU-7 showed higher odds (OR = 1.47; 95% CI = 1.30–1.66) of in-hospital mortality and lower odds (OR = 0.8; 95% CI = 0.72–0.9) of being discharged home after adjustments for co-factors. A longer ICU stay was also associated with higher CAM-ICU-7 scores (*p* = 0.001) [39].

Electroencephalography (EEG), especially spectral analysis, is expected to lead to the development of a simple diagnostic algorithm for the early detection of ICU patients at risk of delirium. An increase in delta power in frontal, central, or temporal regions alone, or in combination with a reduction in beta frequencies in occipital regions measured by only two electrode derivations, showed a high sensitivity and specificity [40]. Other instrumental devices have also attempted to detect delirium, such as continuous non-invasive eye tracking [41].

## 8. Prevention and Treatment of SAD

The prevention and treatment of SAD include non-pharmacological and pharmacological approaches. Although both have a number of risks, clinically modifiable risks are the target of our intervention.

### 8.1. Non-Pharmacological Approach

In view of the prevention of delirium, it is important to remove several risk factors. In addition to age, dementia, emergency surgery, and severity, hypertension, mechanical ventilation, metabolic acidosis, delirium on the previous day, and coma, which may be controlled with active interventions, have been identified as risk factors [42]. The removal of hydration, sepsis, and unnecessary daily medication as risk factors is particularly important for ICU patients in addition to the overuse of benzodiazepines, propofol, opioids, dexmedetomidine for sedation, and painkillers for septic shock patients. The main aim is to maintain mechanical ventilation in ICU patients. Regarding active treatment, early physical mobilization reduced the duration of delirium in 200 patients [43]. Another randomized controlled trial (RCT) showed that early physical occupational therapy for ventilated patients also reduced the duration of delirium [44]. Patients in the physical therapy group had a shorter duration of delirium (median 2.0 days, *p* = 0.02) and more ventilator-free days (23.5 days, *p* = 0.05) during a 28-day period. The ABCDEF bundle in combination with early mobilization may improve survival, delirium, mechanical ventilation, and other factors [45]. In a recent RCT on the effectiveness of combined non-pharmacological intervention therapy including periodic reorientation, correction of sensory deficits, sleep promotion, and cognitive stimulation, the incidence of delirium was lower in the intervention group than in the standard care group [46].

### 8.2. Pharmacological Approach

A recent systematic review indicated that the prevalence of delirium was lower in critically ill patients treated with dexmedetomidine, which is suggested to exert anti-inflammatory effects, than in those treated with placebo and benzodiazepines [47]. However, in an RCT on mechanically ventilated patients with sepsis, a post hoc analysis showed that dexmedetomidine did not increase the number of days free from delirium or coma over that in the control group sedated with medications other than dexmedetomidine, such as propofol or midazolam [48]. In another RCT on mechanically ventilated patients with sepsis, dexmedetomidine did not increase the number of days without delirium or coma over that with propofol [49]. These findings are consistent with a previous study that suggested the selection of propofol or dexmedetomidine over benzodiazepines increased the risk of delirium in mechanically ventilated patients [37]. Several RCTs indicated the effectiveness of haloperidol, atypical antipsychotics, statins, ketamine, and melatonin/ramelteon for the prevention of delirium; however, evidence to support this in septic patients is inadequate [37,50].

**Figure 4:**
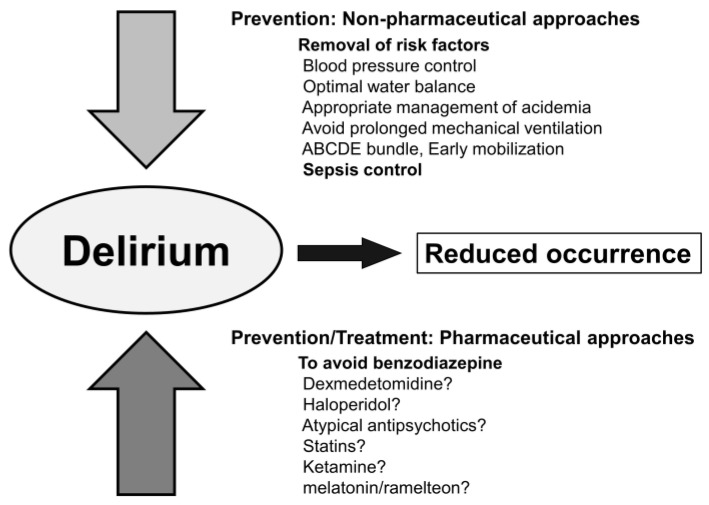
Prevention and treatment of delirium in sepsis-associated delirium.

Few high-quality clinical studies have been conducted on treatments for delirium. An RCT failed to show the benefits of statins for SAD in patients with acute respiratory distress syndrome [51]. In an RCT on agitated delirium in mechanically ventilated patients, the number of ventilator-free hours was significantly higher in patients treated with dexmedetomidine than in those treated with placebo [52]. However, its effectiveness for SAD remains unclear. RCTs on the effects of haloperidol or atypical antipsychotics on delirium failed to demonstrate their benefits [37]. Therefore, there is currently no established pharmacological intervention for the prevention and management of SAD (Figure 4).

## 9. Prognosis of SAD

Delirium affects the prognosis of septic patients. Although there are several confounding factors, delirium in critical care has been associated with higher mortality rates and longer hospital stays [8]. Post-intensive care syndrome (PICS) is currently the focus of research on the long-term prognosis of patients after critical care and consists of three major components: physical impairment, mental disorders, and cognitive dysfunction [53]. The effects of PICS persist for up to several years, and sepsis has been identified as one of the strongest risk factors for PICS [53]. Delirium is considered to correlate with PICS. Among PICS components, cognitive dysfunction was shown to be directly affected by delirium via neural damage. The occurrence of delirium was identified as an independent risk factor for long-term and permanent cognitive dysfunction in ICU patients, including those with sepsis [54]. Limited information is currently available on physical and mental components of PICS. Although delirium may not directly affect physical impairment, it becomes a barrier to early mobilization due to prolonged mechanical ventilation and physical restraint and may also contribute to muscle weakness [55]. Previous studies reported a relationship between delirium and physical impairment [56]. On the other hand, mental disorders, such as anxiety, depression, and post-traumatic stress disorder, were not associated with delirium [57]; however, psychiatric consultation services are offered to ICU patients with the aim of attenuating the mental component of PICS through the management of delirium [58]. Delirium may be regarded as an important factor affecting the outcome of PICS centered on cognitive dysfunction and may be controlled with appropriate assessments and management.

## 10. Delirium in Patients with COVID-19

The difficulties associated with the effective implementation of strategies to prevent and manage delirium in COVID-19 patients remain a major challenge for health-care providers [59]. The most common factors associated with the risk of delirium in COVID-19 patients include hypoxia, coagulopathy, exposure to sedative and analgesic drugs, isolation, and immobility [60]. Furthermore, the strict social isolation of patients has resulted in the more inadequate implementation of the ABCDEF bundle, some of which could also not be implemented frequently before pandemic, which has exaggerated the difficulty of controlling delirium [59,60,61,62].

In a systemic review, the prevalence of delirium at presentation was 24.3% in COVID-19 patients [14]. In a multicenter cohort study on patients aged 65 years and older, 37% with delirium at presentation did not have fever or shortness of breath [63]. Therefore, it is important to note that delirium may be an early symptom of infection or the only symptom in COVID-19 patients presenting to hospitals [59,63]. The incidence of delirium during hospitalization was estimated to be 32.4% [14] and was high at 55–79.5% in ICU patients [60,62,63]. Furthermore, the risk of death was threefold higher in patients with than in those without delirium [14].

Risk factors for delirium include mechanical ventilation, the use of benzodiazepine sedatives, environmental factors, such as immobility and isolation, age > 75 years, and multiple comorbidities [59,63,64]. According to this evidence, practical measures to prevent and manage SAD should be implementing the ABCDEF bundle [60,62]. Social isolation in COVID-19 may be alleviated by allowing family visits with virtual or face-to-face means [65]. Quetiapine and dexmedetomidine have demonstrated their efficacy in the treatment of delirium in COVID-19 [61]. Delirium in severe COVID-19 patients was found to persist for twice as long as in general ICU patients; therefore, it may worsen long-term outcome [60].

## 11. Future Directions

There are currently no specific biochemical tests or treatments for SAD. Delirium by itself not only worsens the long-term prognosis of patients, but it is also regarded as an important factor affecting the outcome of PICS, particularly cognitive dysfunction, and may need to be controlled with appropriate assessments and management, which may be more important in the setting of sepsis. Furthermore, particularly for COVID-19 patients, the difficulty of adequately implementing the ABCDEF bundle and the need for social isolation are issues that require the development of conventional care for SAD. While the early detection and treatment of sepsis, the appropriate use of sedatives, and continued -orientation exercises remain the most effective approaches in the management of patients with SAD, a more detailed understanding of bidirectional signaling pathways between the immune system and brain may lead to the development of future specific therapies.

## 12. Conclusions

Sepsis is a significant risk factor for delirium and is associated with increased mortality, morbidity, and PICS; delirium also represents a specific risk in COVID-19 patients. Further studies are required on the underlying mechanisms, risk factors, prevention, and treatments for SAD.

## Figures and Tables

**Table 1:** Risk factors for sepsis-associated delirium.

Risk Factors for SAD or SAE	
Background	Age ≥ 65 years
	Cognitive dysfunction
	Long-term use of psychoactive drugs
	History of chronic alcohol abuse
Original disease	*Staphylococcus aureus* bacteremia
	Neurological disease
Laboratory tests	Decreased Alb
	Renal failure
	Hypo-/hyperglycemia
	Hypernatremia
	Hypercapnia
Physical examination	Tachypnea
	Low level of consciousness
	Dependent activity and high nursing needs
	Use of vasopressors/inotropes

## Data Availability

Not applicable.
